# A Hybridized Machine Learning Approach for Predicting COVID-19 Using Adaptive Neuro-Fuzzy Inference System and Reptile Search Algorithm

**DOI:** 10.3390/diagnostics13091641

**Published:** 2023-05-06

**Authors:** Thandra Jithendra, Shaik Sharief Basha

**Affiliations:** Department of Mathematics, School of Advanced Sciences, Vellore Institute of Technology, Vellore 632014, India

**Keywords:** coefficient of determination, COVID-19 influenza, fuzzy logic, nature-inspired algorithms

## Abstract

This research is aimed to escalate Adaptive Neuro-Fuzzy Inference System (ANFIS) functioning in order to ensure the veracity of existing time-series modeling. The COVID-19 pandemic has been a global threat for the past three years. Therefore, advanced forecasting of confirmed infection cases is extremely essential to alleviate the crisis brought out by COVID-19. An adaptive neuro-fuzzy inference system-reptile search algorithm (ANFIS-RSA) is developed to effectively anticipate COVID-19 cases. The proposed model integrates a machine-learning model (ANFIS) with a nature-inspired Reptile Search Algorithm (RSA). The RSA technique is used to modulate the parameters in order to improve the ANFIS modeling. Since the performance of the ANFIS model is dependent on optimizing parameters, the statistics of infected cases in China and India were employed through data obtained from WHO reports. To ensure the accuracy of our estimations, corresponding error indicators such as RMSE, RMSRE, MAE, and MAPE were evaluated using the coefficient of determination ($R^{2}$). The recommended approach employed on the China dataset was compared with other upgraded ANFIS methods to identify the best error metrics, resulting in an $R^{2}$ value of 0.9775. ANFIS-CEBAS and Flower Pollination Algorithm and Salp Swarm Algorithm (FPASSA-ANFIS) attained values of 0.9645 and 0.9763, respectively. Furthermore, the ANFIS-RSA technique was used on the India dataset to examine its efficiency and acquired the best $R^{2}$ value (0.98). Consequently, the suggested technique was found to be more beneficial for high-precision forecasting of COVID-19 on time-series data.

## 1. Introduction

COVID-19 is an infectious disease caused by a coronavirus that affects the human lungs and causes severe acute respiratory syndrome (SARS), which can be fatal [1]. The first confirmed case of COVID-19 was diagnosed in Wuhan, China, at the end of December 2019. However, the COVID-19 pandemic was not the first to affect individuals around the world [2]. The virus was initially transferred between civet cats to humans in 2002 [3]. In 2003, a SARS epidemic was first identified, followed by the Middle East respiratory syndrome (MERS) pandemic in 2012. The current COVID-19 pandemic has now presented a novel challenge to the healthcare system and government agencies [2]. It is now known beyond doubt that the coronavirus is transmitted primarily among people who are in close proximity. The diffusion of the virus occurs via aerosol droplets emitted from an infected person’s mouth or nose when they cough, sneeze, speak, sing, or breathe. Touching surfaces that have been contaminated by the virus can also lead to infection if a person subsequently touches sensitive body parts such as the eyes, nose, or mouth. By December 2021, more than 27 million positive cases had been recorded worldwide, with a 98% recovery rate. During the first wave of COVID-19, more than 500,000 daily positive cases were reported worldwide, while the second wave of COVID-19 has caused more than 800,000 daily active cases.

COVID-19 has had an influence on every nation around the globe; some of the countries that have been worst affected by COVID-19 include the United States, India, Brazil, and the United Kingdom, among others. India has been one of the most adversely affected countries in the COVID-19 pandemic, exceeding 44.6 million positive cases and about 536,766 confirmed fatalities by April 2023. India was ranked fifth globally for its infection fatality ratio, and second in the world regarding its recovery rate. The true burden of the disease is unknown due to observational constraints. The maximum number of cases reported by both North and South America indicated that India, specifically, has been significantly impacted. Therefore, complete worldwide eradication is still likely a long way off. Airports, schools, universities, public transportation, industries, and many companies were forced to close as a consequence of the global pandemic [2]. Furthermore, less industrial activity has affected the global economy [4]. Many governments have implemented programs and services to promote awareness about the global pandemic by enacting strict laws and regulations to mitigate the economic repercussions [5]. As a result, we should anticipate that fluctuations in the number of COVID-19 positive cases are likely to continue for the foreseeable future [6].

### 1.1. Research Gap

Deep learning (DL) is a specialized machine learning (ML) technique that uses artificial intelligence (AI) and multiple hidden layers in a neural network. They have played a prominent role in medical sciences, especially in disease prediction. Data science and AI are becoming extremely relevant and useful across the entire healthcare sector. Healthcare experts have been striving to create advanced technologies with automated systems that may be used to resolve problems that arise in this arena. ML has aided in the exact detection of infectious diseases, allowing patients’ illnesses to be diagnosed early. Many scientists are still working on ML techniques for the automatic detection and monitoring of diseases. Support vector machines (SVM), linear regression, logistic regression, and neural networks (NN) can all be used as prediction models. These have recently been used to forecast future occurrences of pandemics [7]. The following are some of the DL approaches used by researchers: Hamid Reza Niazkar et al. developed 14 artificial neural network (ANN) models to estimate a daily number of verified disease cases [8]. Iftikhar Ahmad et al. estimated COVID-19 cases in Pakistan by employing ANN and a strategy based on rectified linear units (RLU) [9]. Tamang et al. estimated COVID-19 cases using an ANN curve-fitting technique [10]. To predict daily positive cases and the mortality of COVID-19 in Brazil, statistical modeling using Weibull distribution was implemented by Vitor Hugo Moreau [11]. Mathematical models evolved by Majid Niazkar et al. were used to assess the COVID-19 spread in Iran and Turkey [8]. To assess COVID-19 based on time-series data, an SVM was implemented by Vijander Singh [12]. Lei Zhang et al. utilized long short-term memory (LSTM) networks to estimate the spread of COVID-19 in Canada [13]. Sunitha et al. implemented Andrew plot-based visualization techniques to predict the onset of pandemics [14]. Mohamed Marzouk et al. implemented AI models, such as convolution neural networks (CNN), LSTM networks, and multi-layer perceptron neural networks, to predict COVID-19 transmission in Egypt [5].

### 1.2. Objectives and Contributions

In this research, we propose an efficient and accurate forecasting model for quickly and precisely forecasting COVID-19 cases. Fuzzy logic, neural networks, and meta-heuristic (MH) computation have been demonstrated as effective in the diagnosis of disease [15]. One of the best-known approaches for these predictions using AI is the ANFIS model. Soft-computing techniques, such as optimization and forecasts, have been implemented for a variety of real global issues and used to tackle complex problems, such as diagnosing diseases, predicting stock market prices, anticipating electricity usage, etc., on a worldwide scale. Jang [16] invented ANFIS in 1993, which has become one of the most extensively used neuro-fuzzy systems. However, researchers have discovered numerous optimal strategies to update the premise and the consequent parameters for strengthening the ANFIS models. Obtaining the model parameters is a challenging problem to handle nonlinear problems using ANFIS. In order to solve this issue, previous research has shown that using evolutionary approaches for parameter selection was more effective. Table 1 illustrates how several nature-inspired meta-heuristic strategies were incorporated into various ML models. As shown in Table 1, meta-heuristic optimizations (MHO) have been effective in identifying optimal solutions for many difficult optimization problems. Training ANFIS is also regarded as one of the most challenging optimization issues. Thus, in previous works, ANFIS has been taught to recognize non-linear static and dynamic systems using meta-heuristic algorithms.

In this study, the RSA developed by Abualigah et al. [17] in 2022, is used to estimate parameter values of ANFIS. The RSA algorithm uses crocodile hunting techniques: exploration (global search) and exploitation (local search). The exploration strategy relates to crocodile belly-walking and high-walking, whereas the exploitation strategy refers to hunting coordination and cooperation [17]. Based on earlier research, the RSA technique was improved and added to ANFIS to select the ideal parameters and improve the model’s performance.

**Table 1:** Different meta-heuristic strategies were interpolated to advance AI models.

AI Model and MH Algorithm	Acquired Results	Ref.
Artificial Neural networks (ANN) Genetic Algorithm (GA)	Financial data mining instances identification and forecasting of imbalanced datasets, reliability, and extraction features.	[18]
ANN and Grey Wolf Optimizer	Modeling COVID-19 using ANN-GWO with MAPE for training (6.23), testing (13.15), and Validation (11.4).	[19]
ANN and PSO	To transfer the particles to the new best-predicted destination, PSOCoG is implemented as the ‘Center of gravity’.	[20]
ANFIS and Virus Optimization Algorithm	Estimated the influence of several parameters on the infection rate and performed a regression analysis that was successful.	[21]
ANFIS and Beetle Antennae Search (BAS)	Improving ANFIS parameters and anticipating COVID-19 positive instances using the Beetle Search technique.	[2]
ANFIS and mutation-based Bees Algorithm (mBA)	employed the mBA strategy to improve ANFIS parameters and diagnose COVID-19.	[6]
FPASSA-ANFIS	Modified the FPA with the assistance of SSA in order to improve the ANFIS to the anticipation of COVID-19.	[22]

Then, ANFIS-RSA, a system based on ANFIS and trained using the RSA, was used to create a model for predicting the COVID-19 outbreak. This is the first work to develop a prediction model using ANFIS and RSA for COVID-19 outbreak analysis. The primary objective of this research is to improve the accuracy of future COVID-19 infection forecasts. Since COVID-19 is by far the most critical and significant global catastrophe that humanity has ever faced, the recommended technique has been tested on the COVID-19 data collected from China and India. The investigation is conducted using a huge dataset for China (21 January 2020, to 18 February 2021) obtained from [2] and India (3 November 2020, to 1 January 2022) retrieved from World Health Organization (WHO). Following that, ANFIS-RSA assesses each dataset using the training set, which consists of 75% of the total data. A testing dataset (25%) is used to assess the performance of the trained ANFIS-RSA model. The authors compared ANFIS-RSA to previous research [2,22], which is shown in Table 2. Statistical benchmarks such as mean squared error (MSE), root mean squared error (RMSE), mean absolute error (MAE), mean absolute percentage error (MAPE), root mean square relative error (RMSRE), and determination coefficient ($R^{2}$) were used to show how well the proposed model works and how accurate it is. In comparison to these simulations- and experiment-tested models, ANFIS-RSA has the lowest statistical measures and the best accuracy. The experimental results indicate that the ANFIS-RSA integrated approach can provide an estimate of COVID-19 cases that is more precise than either of the techniques alone.

The scientific achievements are highlighted and summarised as follows:

- To develop an effective time-series forecasting model for the COVID-19 epidemic utilizing an ANFIS model and a reptile search algorithm.
- The proposed ANFIS-RSA enhances the effectiveness of the conventional ANFIS model and delivers considerable results in comparison to previous studies.
- We have implemented the ANFIS-RSA AI model to the COVID-19 data of China and compared the obtained error metric results with different optimizing algorithms utilized in ANFIS models. Furthermore, we have predicted a recent diffusion of COVID-19 in India in 2022 to test the validity of the trained model.
- In comparison to other models, the ANFIS-RSA has the best statistical measures, including RMSE (3196), MAE (1550), MAPE (0.0672), RMSRE (0.0962), and $R^{2}$ (0.9775) for China. Similarly for India, ANFIS-RSA achieved RMSE (8921.608), MAE (4570.359), MAPE (0.2252), RMSRE (0.3338), and $R^{2}$ (0.9874).
- According to the experimental findings, it is strongly recommended that the ANFIS-RSA hybridized model be used to improve the exactness of the forthcoming epidemic estimations.

The remaining sections of this paper are arranged as follows: In Section 2, a brief review of the literature is given. The data collection, ANFIS insights, and reptile search method are provided in Section 3. Section 4 highlights the proposed model, ANFIS-RSA. The experimental results and discussion of the model are included in Section 5, while the conclusion is presented in Section 6.

## 2. Literature Review

The section offers a thorough investigation of the ANFIS model and meta-heuristic algorithms used in AI models to solve some of the most prevalent global challenging problems. Jang designed the Adaptive Neuro-Fuzzy Inference System, a soft computing technology that consists of neural networks and fuzzy logic, in 1993 [16], and it has since been used in a broad range of real-world applications, engineering science, and earth sciences [23]. ANFIS was the hybridized version of neural networks and fuzzy inference systems that come under the conglomerate of artificial intelligence approaches [2]. As evidenced by the extent of past research, ANFIS has been implemented in time series predictions including healthcare systems, image analysis, data extraction, and classification, the prognosis of diseases, and so on [24]. To optimize the premise and consequent parameters, a hybrid learning strategy was used, which integrates gradient descent and the least squares approach [25]. ANN models use connected neurons to mimic human intelligence [26].

The artificial intelligence prediction models ANFIS, feed-forward neural network (FFNN), support vector machine (SVM), and multi-linear regression (MLR) have been presented by Abegaz and Etikan [27] to evaluate the mortality of COVID-19 in East Africa. In their analysis, the ensemble ANFIS outperformed linear techniques in terms of accuracy. When the forecasting precision as compared to the marine predator algorithm (MPA) and particle swarm optimization (PSO) algorithms, it was found that the presented model performed better. Abunadi et al. [28] developed GSO-IDCNN, a novel combination of an inception-based deep convolutional neural network and glow-worm swarm optimization, for the detection and classification of COVID-19. A novel COVID-19 prediction model that incorporates the chaotic marine predator algorithm and ANFIS is presented by Al-qaness et al. [29]. On the other hand, Nayak et al. [30] looked into the effects of COVID-19 on a wide range of industries, such as transportation, electricity and power, agriculture, education, travel and tourism, and consumer technology. Recently, Ozturk et al. [31] designed a neuro-fuzzy inference system based on genetic algorithms to identify between benign and malignant thyroid nodules. Ref. [32] performed a state-of-the-art evaluation of the current ML and DL methodologies in the identification and prediction of COVID-19. For diagnosing cardiac problems, Mohammad Ayoub Khan and Fahad Algarni [33] used the technique named Modified Salp Swarm Optimization (MSSO) called MSSO-ANFIS.

The most difficult aspect of ML and AI technologies is tweaking the parameters and determining the best solution to accomplishing challenging problems. In recent research, nature-inspired meta-heuristic algorithms have played an important role in finding the best solution. Among the most well-known kinds of meta-heuristics are bio-inspired algorithms. These are divided into two categories: (i) evolutionary algorithms, and (ii) swarm intelligence algorithms. We looked at different meta-heuristic algorithms and how they have been used in ANFIS to optimize parameters and identify the optimum solution.

An overview of several evolutionary algorithms and their application in ANFIS for prediction are as follows. Goldberg’s Genetic Algorithm (GA) was the well-known evolutionary approach devised in 1989 [34] and has since been utilized to tackle global issues. It was used by Liang-YingWei in ANFIS to estimate national stock market swings in 2013 [35]. The particle swarm optimization (PSO) technique was one of the earliest algorithms presented in the field of swarm-based searching [36]. PSO-ANFIS was used to assess the demand for energy in industrial domains, and it has been utilized in various real-world problems [37]. One of the most well-known Ant Colony Optimization (ACO) techniques [38] that was used in ANFIS to evaluate mammography images [39] and was inspired by real-life ant behavior. Another swarm strategy known as Cuckoo Search (CS) [40] has been successfully used in crude price estimation and to improve AI models [41]. By influencing the bearing of fireflies and their illuminating features the approach was called Firefly Algorithm (FA) and widely used in enhancing the ML models [42]. Elephant herding optimization (EHO) was inspired by the behavior of a herd of elephants that were used to solve a variety of complex problems [43]. Tree Growth Algorithm (TGA) [44], Brain Storm Optimization (BSO) [45], Beetle Antennae Search (BAS) [46] and many other algorithms are used for tackling complex problems. Researchers used meta-heuristic methodologies in the ANFIS model to diagnose the global challenge of COVID-19 Influenza in recent studies. Case in point, ANFIS-VOA (Virus Optimization Algorithm) is developed to assess the risk of COVID-19 dissemination in the US [21]. In order to anticipate COVID-19 confirmed cases, the ANFSI-mBA (mutation-based Bees Algorithm) approach was used in the United States and India [6]. Al-qaness et al. [22] developed a combination of FPA and SSA algorithms called FPASSA to estimate the COVID-19 cases in China. Due to COVID-19 lockdowns, the ANFIS model was enhanced by including the PSO algorithm for assessing the quality of air in Wuhan, China, and obtained the finest results [47]. ANFIS-BAS (Beetle Antennae Search) and ANFIS-CESBAS (Cauchy Exploration Strategy Beetle Antennae Search) algorithms attained superior results in order to predict COVID-19 in China [2].

## 3. Materials and Methods

### 3.1. Data Collection

**Figure 1:**
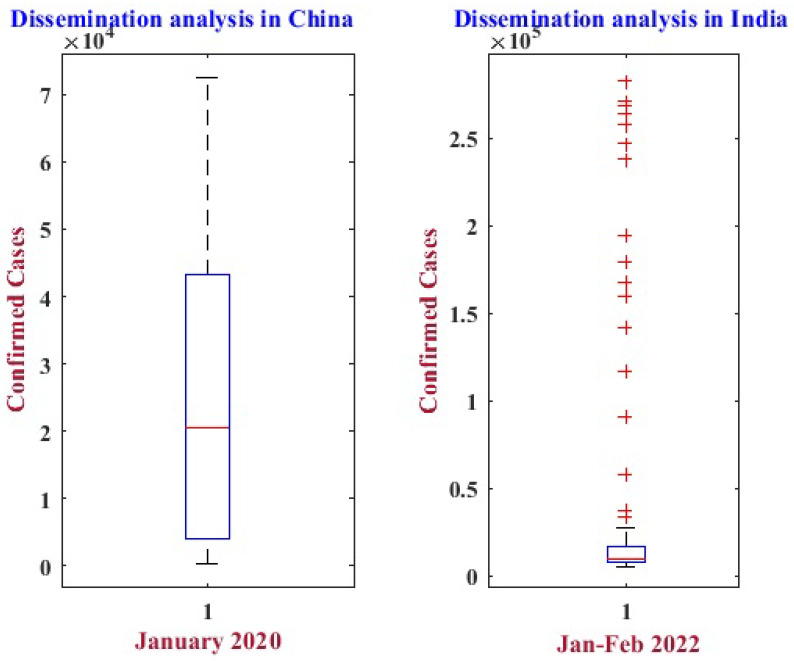
Dissemination analysis of confirmed cases in China and India.

This study considered the exploratory data of COVID-19-positive cases from China in January 2020, as well as recent data from India around January and February 2022, in order to enhance the anticipated precision. To compare the forecast outcomes, data from China was used from Miodrag Zivkovic [2]. The COVID-19 dataset for India was collected from the official site of the World Health Organization [48]. Figure 1 presents COVID-19’s analysis of the lowest, median, and highest dispersion throughout China and India.

### 3.2. Adaptive Neuro-Fuzzy Inference System

**Figure 2:**
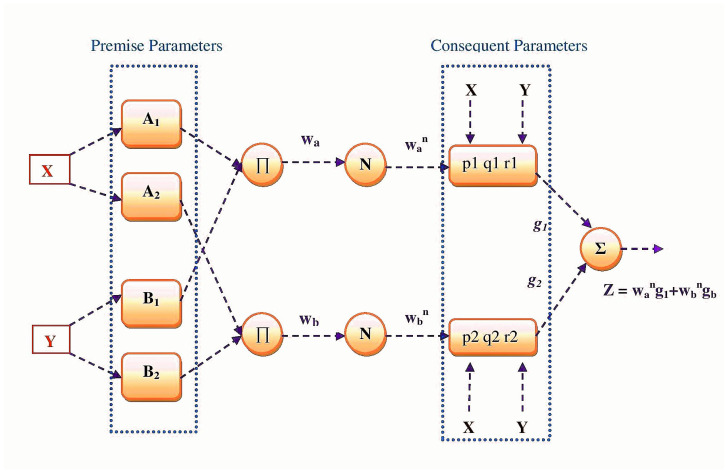
Architecture of ANFIS model.

The ANFIS is a five-layered hybrid network composed of fuzzy logic and neural networks. With the goal of improving decision-making in the face of unclear, inaccurate, and inconsistent data, Zadeh [49] came up with the concept of fuzzy logic and fuzzy inference systems in 1965. The function of brain neurons served as the inspiration for Warren McCulloch and Walter Pitts’ 1943 invention of neural networks, also known as “connectionism”, which refers to the use of connected neurons to mimic human intelligence [26]. In the last decade, the Neuro-Fuzzy system, which combines neural networks with fuzzy logic, has been widely used to simulate non-linear issues and global concerns. Jang [16] developed the Adaptive Neuro-Fuzzy Inference System (ANFIS) in 1993 as a neuro-fuzzy system that integrates the capabilities of modeling neural networks with fuzzy logic to mimic an expert decision-making process. As part of ANFIS, ANN’s learning capacity and spatial structure are integrated with fuzzy logic’s decision-making process. Similar to ANNs, ANFIS implements learning with samples based on a training database [24]. ANFIS modeling employs the Takagi-Sugeno fuzzy system, which consists of two stages: premise and consequence. The ANFIS model consists of five layers, as shown in Figure 2: fuzzification, product, normalization, defuzzification, and output.

**Layer1:** In layer 1, membership functions are used to change input values from crisp to fuzzified values, and all nodes are adaptable. The output in this layer is computed using Equation (1).

(1) $${O_{i}}^{1}=\mu_{A_{i}}\left(x\right),\;i=1,2$$

(2) $${O_{i}}^{1}=\mu_{B_{i}}\left(y\right),\;i=1,2$$

Nodes were designated as $A_{1},A_{2},B_{1},$ and $B_{2}$. Equations (1) and (2) were used to get fuzzified values for the inputs. Due to its efficiency, the Sigmoidal MFn has been adopted for this research and denoted as $f_{A_{n}}\left(x\right)$.

(3) $$\mu_{A_{i}}\left(x\right)=Sigmoid\left(x;a,c\right)=\frac{1}{1+exp^{-a(x-c)}}$$

**Layer2:** In layer 2, each node’s output is computed by multiplying its input connections using fuzzy AND. At this step, nodes are categorized by π and Equation (4) is used to compute their output.

(4) $${O_{i}}^{2}=w_{i}=\mu_{A_{i}}\left(x\right)*\mu_{B_{i}}\left(y\right),\;i=1,2$$

**Layer3:** In layer 3, the fuzzy strengths of the second layer are utilized to determine normalized values, and the nodes of this layer are designated *N*. The normalization procedure indicates the ratio of the current firing strength to the overall firing strength across all rules, and nodes cannot be modified during this phase. Equation (5) is used to derive the solution for this layer, which is discussed below.

(5) $${O_{i}}^{3}=\bar{w_{i}}=\frac{w_{i}}{\sum_{i=1}^{2}w_{i}},\;i=1,2$$

**Layer4:** In layer 4, defuzzification methods are used to transform fuzzy outputs into crisp outputs. At this level, nodes are adaptive with a node function. As a result, the output is estimated by multiplying the previous output by the linear equation, as given in Equation (6).

(6) $${O_{i}}^{4}=\bar{w_{i}}*f_{i}=\bar{w_{i}}*\left(p_{i}\left(x\right)+q_{i}\left(y\right)+r_{i}\right),\;i=1,2$$

Here, $\bar{w_{i}}$ is the normalized output and $p_{i},q_{i},r_{i}$ is the parameter in this stage.

**Layer5:** In layer 5, it is proved that the nodes are not adaptive and that the output is derived by aggregating the outputs of the previous levels, as indicated in Equation (7).

(7) $${O_{i}}^{5}=\sum_{i}\bar{w_{i}}*f_{i}$$

Generally in the ANFIS model, least squares and gradient descent methods were used for upgrading the parameters in the second and fourth layers. We incorporated the RSA in the ANFIS model to improve the parameters and make the ANFIS model more efficient based on our previous expertise with several evolutionary strategies.

### 3.3. Reptile Search Algorithm (RSA)

Abualigah et al. invented the RSA in 2022, which is a nature-inspired meta-heuristic algorithm [17]. The meta-heuristic technique was motivated by the predatory strategy of crocodiles, more exactness by foraging for food. The heuristic was further split into two crocodile hunting strategies: exploration and exploitation. Crocodile behavior was split into two categories in terms of exploring strategy: (i) high walking and (ii) belly walking. Regarding exploitation strategy, hunting coordination and collaboration are taken into account. Between the exploration and exploitation foraging stages, the RSA algorithm was altered. The total number of iterations was split into four sections based on these methodologies. The exploration strategy met two conditions: high walking $\left(t\leq \frac{T}{4}\right)$ and belly walking $(t>\frac{T}{4}$ and $t\leq \frac{2T}{4})$. Moreover, the exploitation strategy was conditioned by hunting coordination $(t>\frac{2T}{4}$ and $t\leq \frac{3T}{4})$ and hunting cooperation $(t>\frac{3T}{4}$ and t≤T) (Abualigah et al.). On real-world optimization problems, this comparatively recent metaheuristic already has achieved extremely positive prospects. The RSA algorithm’s step-by-step methodology can be summarized as follows:

The RSA algorithm is implemented by starting with solutions chosen at random and generating them as

(8) $$y_{\left(i,j\right)}=rand\times \left(UB-LB\right)+LB,\;j=1,2,\cdots ,n$$

In the encircling phase, updating the solution’s position is specified as

(9) $$y_{\left(i,j\right)}\left(t+1\right)=\{\begin{array}{cc} Best_{j_{1}}\left(t\right)\times -\eta_{1}\left(t\right)\times \beta_{1}-R_{1(i,j)}\times rand, & t\leq \frac{T}{4}\\ Best_{j_{1}}\left(t\right)\times x_{\left(r_{1},j\right)}\times ES_{1}\left(t\right)\times rand, & t>\frac{T}{4}\;and\;t\leq \frac{2T}{4} \end{array}$$

The previous best solution achieved is $Best_{j_{1}}\left(t\right)$, while rand signifies a random number between 0 and 1. Furthermore, $\beta_{1}$ is a crucial parameter that affects the exploratory performance, whereas *t* and *T* reflect the current and total number of iterations. ES(t) is the stochastic value among −2 and 2 across the whole iterations evaluated by using Equation (10), and $x_{\left(r_{1},j\right)}$ is the arbitrary position of *i*th solution. $R_{1(i,j)}$ is diminished search area that is computed using Equation (12), $r_{1}$ is the random choice lying in [1N], where *N* is the total number of solutions and Equation (11) is used to calculate $\eta_{1(i,j)}$. It defines the hunting operator to the *j*th position of *i*th solution.

(10) $$ES_{1}\left(t\right)=2\times r_{3}\times \left(1-\frac{1}{T}\right)$$

(11) $$\eta_{1(i,j)}=Best_{j_{1}}\left(t\right)\times P_{1(i,j)}$$

(12) $$R_{1(i,j)}=\frac{Best_{j_{1}}\left(t\right)-x_{\left(r_{2},j\right)}}{Best_{j_{1}}\left(t\right)+\epsilon }$$

Here ϵ represents a small value, $r_{2}$ belongs to [1,N], and $r_{3}$ signifies the arbitrary value in [−1,1]. In the RSA algorithm’s exploitation phase, we utilized Equation (13) to calculate the new solution.

(13) $$y_{\left(i,j\right)}\left(t+1\right)=\{\begin{array}{cc} Best_{j_{1}}\left(t\right)\times P_{1(i,j)}\times rand, & t>\frac{2T}{4}\;and\;t\leq \frac{3T}{4}\\ Best_{j_{1}}\left(t\right)-\eta_{1(i,j)}\left(t\right)\times \epsilon -R_{1(i,j)}\times rand, & t>\frac{3T}{4}\;and\;t\leq T \end{array}$$

$P_{1(i,j)}\left(t\right)$ is defined as the discrepancy of percentage determined by Equation (14) among *j*th place of the best one and *j*th place of the current one.

(14) $$P_{1(i,j)}\left(t\right)=\alpha +\frac{x_{\left(i,j\right)}-M_{1}\left(x_{i}\right)}{Best_{j_{1}}\left(t\right)\times \left(UB_{j}-LB_{j}\right)+\epsilon }$$

where α, another parameter with fixed value 0.1, is used to restraint the exploration precision and $M_{1}\left(x_{i}\right)$ is computed by Equation (15) as

(15) $$M_{1}\left(x_{i}\right)=\frac{1}{n}\sum_{j=1}^{n}x_{\left(i,j\right)}$$

The RSA algorithm’s pseudo code is shown below in Algorithm 1.
**Algorithm 1** Pseudocode of Reptile Search Algorithm.Initialize the parameters randomly $\alpha ,\beta_{1},\epsilon ,$ etc.Setup the candidate solutions: $X=x_{\left(i,j\right)},\;i=1,2,\cdots ,N;\;j=1,2,\cdots ,N$**while** (t<T) **do**Determine the objective function for each of the possible solutions (X).Pick the best solution yetUsing Equation (10), upgrade the ES.**RSA’s begining****for** (i=1toN) **do****for** (j=1ton) **do**Update the values of η, R, *P* by Equations (11), (12), and (13), respectively.**if** ($t\leq \frac{T}{4}$) **then**$y_{\left(i,j\right)}\left(t+1\right)=Best_{j_{1}}\times -\eta_{1(i,j)}\left(t\right)\times \beta_{1}-R_{1(i,j)}\times rand$, ⇒ {highwalking}**else if** ($t>\frac{T}{4}\;and\;t\leq \frac{2T}{4}$) **then**$y_{\left(i,j\right)}\left(t+1\right)=Best_{j_{1}}\times x_{\left(r_{1},j\right)}\times ES_{1}\left(t\right)\times rand$, ⇒ {bellywalking}**else if** ($t>\frac{2T}{4}\;and\;t\leq \frac{3T}{4}$) **then**$y_{\left(i,j\right)}\left(t+1\right)=Best_{j_{1}}\left(t\right)\times P_{1(i,j)}\times rand$, ⇒ {hunting coordination}**else**$y_{\left(i,j\right)}\left(t+1\right)=Best_{j_{1}}\left(t\right)-\eta_{1(i,j)}\left(t\right)\times \epsilon -R_{1(i,j)}\times rand$, ⇒ {hunting cooperation}**end if****end for****end for**t=t+1**end while**Return the best solution (Best(X))

## 4. Proposed Model (ANFIS-RSA)

**Figure 3:**
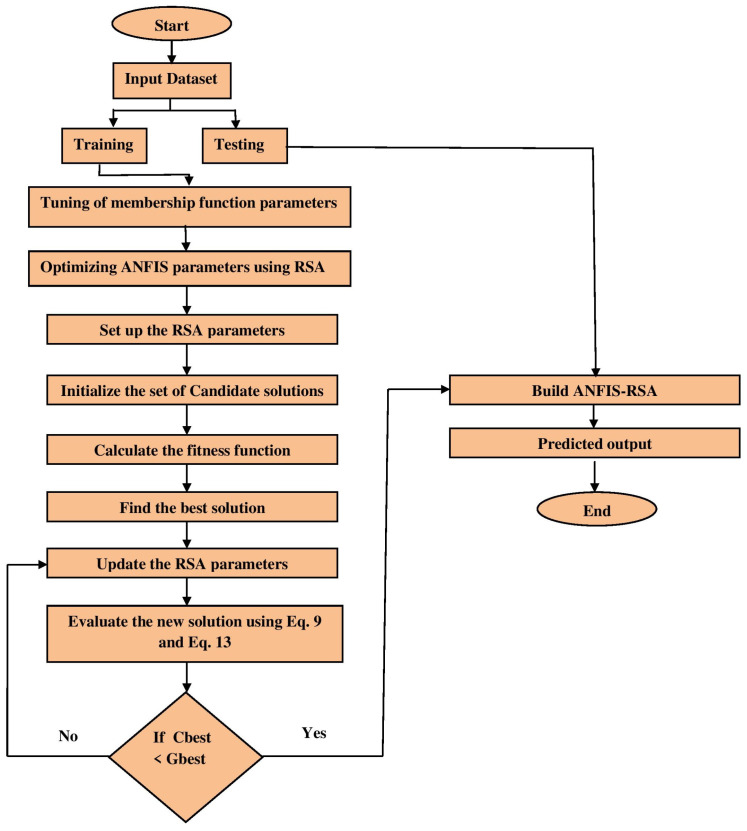
Flow chart of enhanced model ANFIS-RSA.

As the ANFIS parameters have a great impact on the performance and efficiency of the overall system, metaheuristic optimization methods have been used in the past to improve ANFIS time series forecasting. The objective of the proposed method is to enhance the effectiveness of the ANFIS by identifying its parameters using the RSA optimization technique. The hybrid strategy was designated ANFIS-RSA. Generally, the ANFIS training procedure includes improving the system’s design and parameters for a specific problem. The entire number of ANFIS parameters depends on the number of inputs and rules, as well as the kind and a number of membership functions. In ANFIS-RSA, the total number of parameters is identified as the sum of the premise and consequent parameters. The presented model is based on the conventional ANFIS model, which consists of five layers as seen in Figure 2. Layer 1 utilizes input values, whereas layer 5 delivers predicted values. The RSA algorithm is used to estimate the best parameters in ANFIS training. Figure 3 shows the structure of the proposed ANFIS-RSA model for predicting COVID-19 influenza and improving the parameters in the ANFIS model using the RSA technique. At the start of ANFIS-RSA, as seen in Figure 3, it organizes input data for training and testing. Using the train-test splitting method, the model was trained and tested using 75% of the data set for training and 25% for testing. In this study, the sigmoid membership function was considered for fuzzifying the inputs, which was computed using Equation (16). Thereafter, the RSA optimization adjusts ANFIS parameters. In ANFIS-RSA, α and $\beta_{1}$ are defined as the control parameters of RSA in order to get the ideal parameters for ANFIS. In the exploration phase, α=0.1 is used to optimize the exploration efficiency, whereas $\beta_{1}=0.1$ is used to control the exploratory performance in the exploitation phase. Finally, the optimal solution (ANFIS structure) delivered by the RSA is sent to the ANFIS, with which the test phase is performed.

(16) $$f\left(x\right)=\frac{1}{1+exp^{-a(x-c)}}$$

## 5. Experimental Results

The hybridized ANFIS-RSA technique was used to predict future COVID-19 cases by taking into account confirmed cases from China and India. The recommended approach was compared to the outcomes of different MH techniques recorded in Table 2 for the China data set adopted from Zivkovic [2]. In an effort to demonstrate a superior examination of ANFIS-RSA efficacy, additional computations were carried out using datasets from India. To confirm the adequacy of the hybridized model ANFIS-RSA’s predictions, the following five statistical criteria were also evaluated: root mean square error (RMSE), root mean squared relative error (RMSRE), mean absolute percentage error (MAPE), mean absolute error (MAE), and coefficient of determination $\left(R^{2}\right)$. The mathematical representation of the following metrics can be as

(17) $$RMSE=\sqrt{\frac{1}{N_{1}}\sum_{i=1}^{N_{1}}{\left(y_{pred}-y_{actual}\right)}^{2}}$$

(18) $$RMSRE=\sqrt{\frac{1}{N_{1}}\sum_{i=1}^{N_{1}}{\left(\frac{y_{pred}-y_{actual}}{y_{pred}}\right)}^{2}}$$

(19) $$MAPE=\frac{1}{N_{1}}\sum_{i=1}^{N_{1}}\frac{|y_{pred}-y_{actual}|}{y_{pred}}$$

(20) $$MAE=\frac{1}{N_{1}}\sum_{i=1}^{N_{1}}\left|y_{pred}-y_{actual}\right|$$

(21) $$R^{2}=1-\frac{\sum_{i=1}^{N_{1}}{\left(y_{actual}-y_{pred}\right)}^{2}}{\sum_{i=1}^{N_{1}}{\left(y_{actual}-y_{mean}\right)}^{2}}$$

where $y_{pred},\;y_{actual}$ denote forecasted, observed values and $N_{1}$ is the sample size, $y_{mean}$ defines the mean of the actual values. Lower values of RMSE, RMSRE, MAPE, and MAE imply very good efficiency of the model; however, a greater value of $R^{2}$ suggests better performance with high accuracy.

### 5.1. ANFIS-RSA Simulation Using Data from China

**Figure 4:**
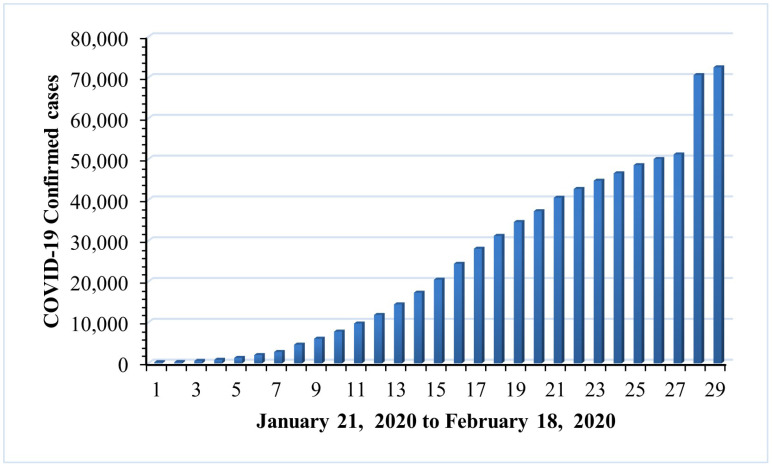
A bar graph depicting the rise in daily cases reported in China.

**Figure 5:**
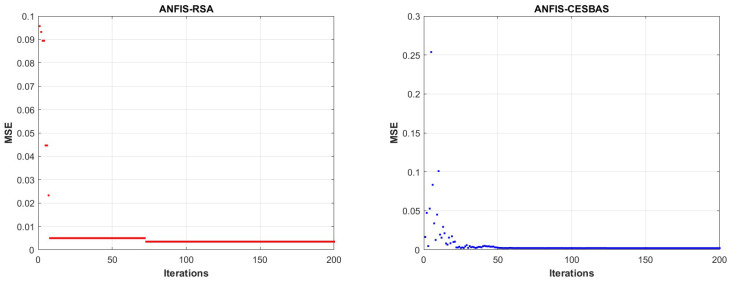
Convergence speed of trained ANFIS-RSA vs. ANFIS-CESBAS models for China.

As part of our research, ANFIS-RSA was run on COVID-19-positive cases from China and made comparative analysis with recently improved ANFIS models: ANFIS-Genetic algorithm, ANFIS-Particle swarm optimization, ANFIS-Beetle antennae search, ANFIS-Flower pollination algorithm ANFIS-ABC, and Cauchy exploration strategy BAS. The data from China includes small anomalies, and we saw a tiny increase in daily confirmed cases in China, as per WHO surveys which is depicted in detail in Figure 4. Figure 5 presents the comparative study of the speed of converging mean squared error (MSE) of the proposed technique ANFIS-RSA and the ANFIS-CESBAS approach. Table 2 includes all of the optimization strategies and comparison studies as well. The results include the best performance identified for different enhanced ANFIS models as well RMSE, RMSRE, MAPE, MAE, and $R^{2}$. As demonstrated by the performance time and the improvement strategy, ANFIS-CESBAS outperformed, although it was still far behind the suggested technique ANFIS-RSA. For instance, the $R^{2}$ value of the ANFIS-CESBAS is 0.9763, whereas the $R^{2}$ value of the ANFIS-RSA is 0.9775.

For the China dataset, the recent approaches ANFIS-CESBAS and FPASSA-ANFIS attempted to provide superior metric values than ANFIS-RSA. However, as shown in Table 2, the suggested method surpassed other approaches in terms of metric values: RMSE (3196), MAE (1550), MAPE (0.0672), and RMSRE (0.0962), with execution time of 20.1 s on a Pentium P5 quad-core laptop. The Table 3 displays our testing results of ANFIS-RSA for India, indicating that ANFIS-RSA also outperforms other models for the test data.

**Table 2:** Comparison of ANFIS-RSA against several upgraded techniques for forecasting COVID-19.

Enhanced Models	RMSE	MAE	MAPE	RMSRE	$R^{2}$	Time	Reference
ANN	8750	5413	13.09	0.204	0.8991	-	[22]
KNN	12,100	7671	8.32	0.130	0.7710	-	[22]
SVR	7822	5354	8.40	0.080	0.8910	-	[22]
ANFIS	7375	5523	5.32	0.09	0.9032	-	[22]
ANFIS-PSO	6842	4559	5.12	0.08	0.9492	24.1	[22]
ANFIS-GA	7194	4963	5.26	0.08	0.9575	27.0	[22]
ANFIS-ABC	8327	6066	6.86	0.10	0.7906	46.8	[22]
ANFIS-FPA	6059	4379	5.04	0.07	0.9439	23.4	[22]
ANFIS-FPPASSA	5779	4271	4.79	0.07	0.9645	23.4	[22]
ANFIS-BAS	7069	5125	6.56	0.10	0.7952	16.6	[2]
ANFIS-CESBAS	4329	3195	4.08	0.06	0.9763	19.8	[2]
**ANFIS-RSA**	**3196**	**1550**	**0.0672**	**0.0962**	**0.9775**	**20.1**	

**Figure 6:**
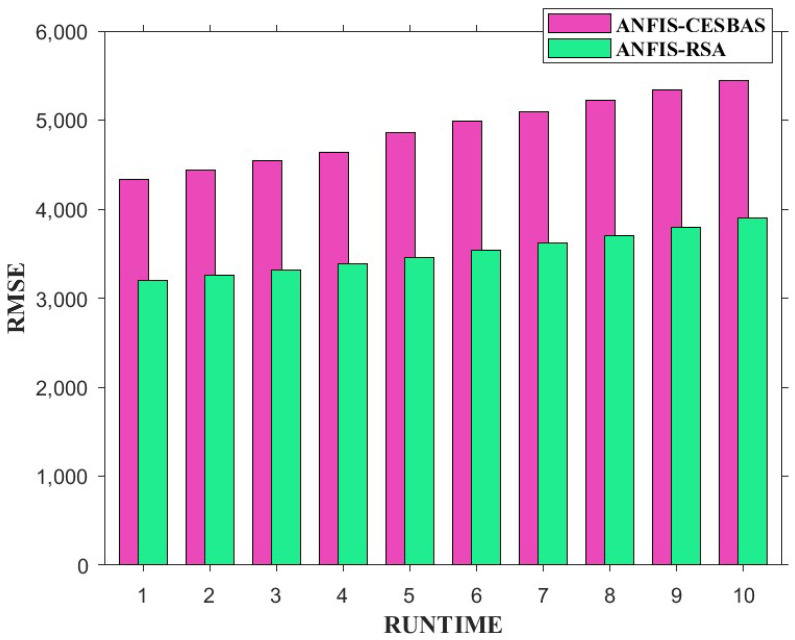
Graphical comparison of ANFIS-RSA and ANFIS-CESBAS in the form of bar plots of RMSE values of 10 independent runs.

**Figure 7:**
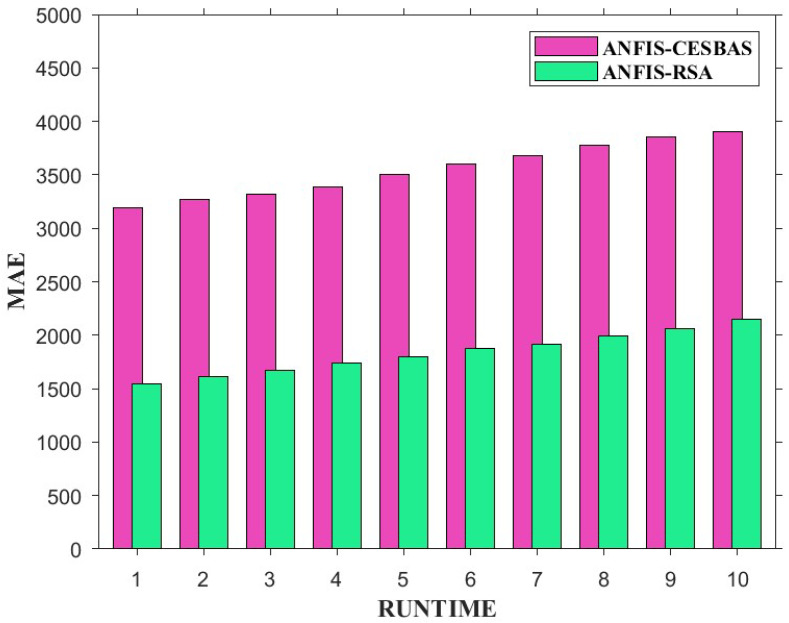
Graphical comparison of ANFIS-RSA and ANFIS-CESBAS in the form of bar plots of MAE values of 10 independent runs.

The suggested approach and ANFIS-CESBAS were run in ten different ways for a thorough comparison; the results were as follows: the first run had the lowest RMSE and MAE, while the final run had the highest RMSE and MAE, resulting in the poorest forecasts owing to the decreasing the number of data. The comparison between both approaches was represented in Figure 6 and Figure 7.

**Figure 8:**
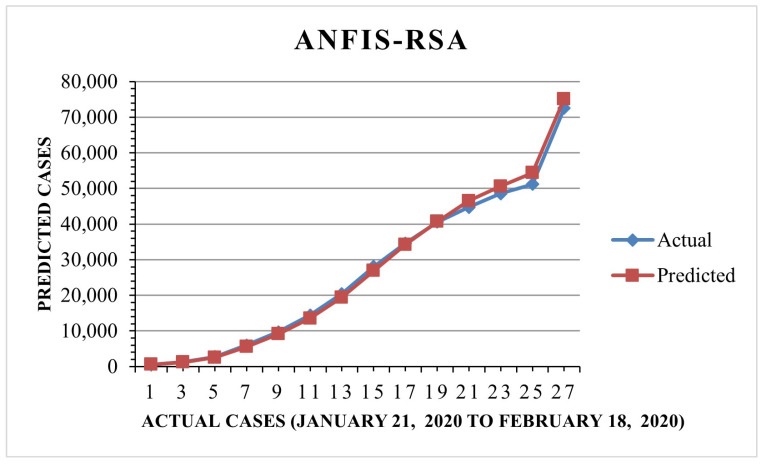
ANFIS-RSA forecast COVID-19 infections for China.

**Figure 9:**
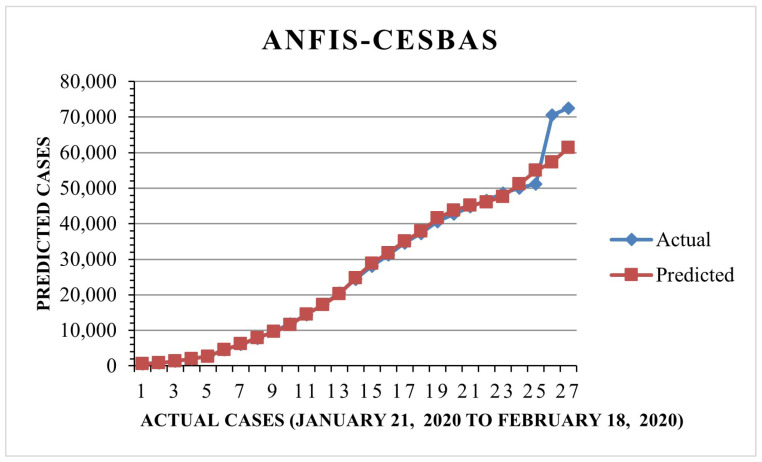
ANFIS-CESBAS forecast COVID-19 infections for China.

**Figure 10:**
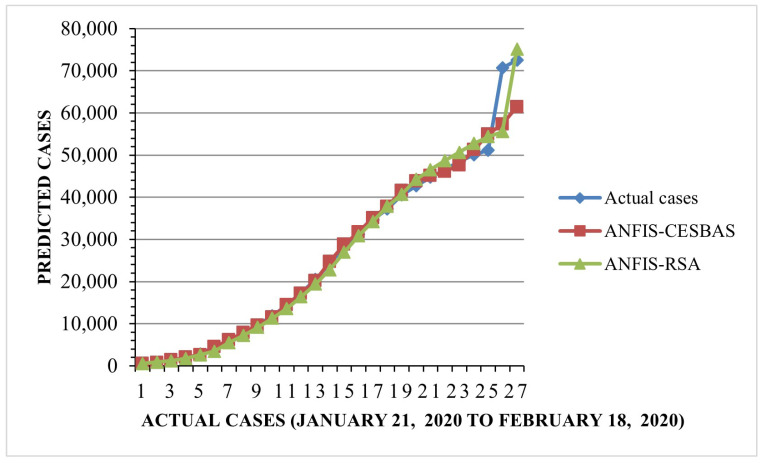
Comparative analysis of ANFIS-RSA vs. ANFIS-CESBAS predictions for China.

Furthermore, we found that the suggested method, ANFIS-RSA, was effective in accurately assessing COVID-19 infections. Figure 8 and Figure 9 show the predictions made using ANFIS-RSA and ANFIS-CESBAS, respectively. Figure 10 depicts the comparative analysis performed with ANFIS-CESBAS.

### 5.2. ANFIS-RSA Simulation Using Data from India

**Figure 11:**
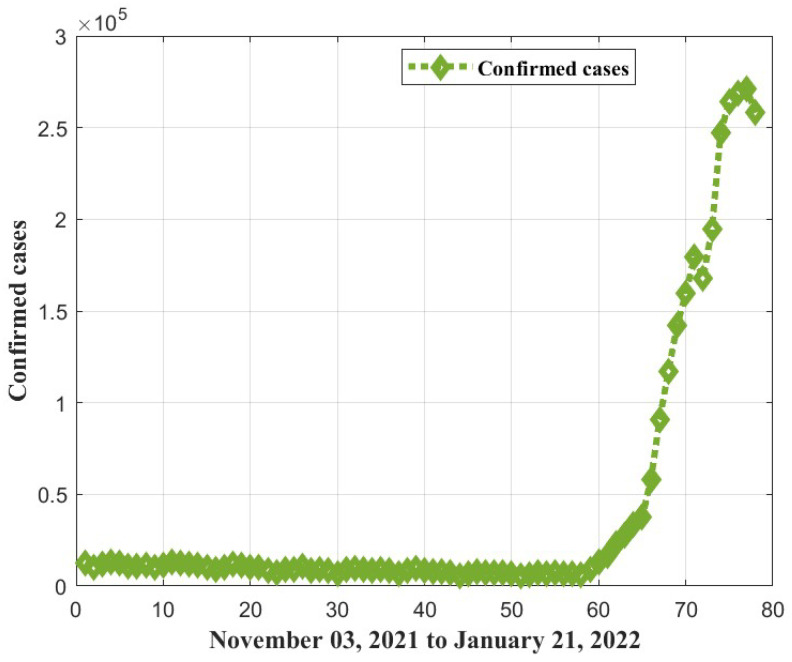
Confirmed COVID-19 cases in India from 3 November 2021 to 21 January 2022.

For our simulation study for India, positive cases from WHO reports between 3 November 2021, and 21 January 2022, were used to assess COVID-19 infections. As can be seen in Figure 11, the dataset fluctuated substantially, and there was a significant rise in positive infections from 1 January 2022 to 21 January 2022. The fluctuations and instant rise of positive cases lead to the inaccuracy of predictions. We explored numerous algorithms, and propose ANFIS-RSA as the best to estimate COVID-19 infections more precisely. Table 4 presents the evaluated metric values and the recommended technique produced significant results when compared to other approaches, with the minimum RMSE (8921.608) and greatest coefficient of determination (0.9874). In Table 5, an ANFIS-RSA model provides the best error value for testing.

**Figure 12:**
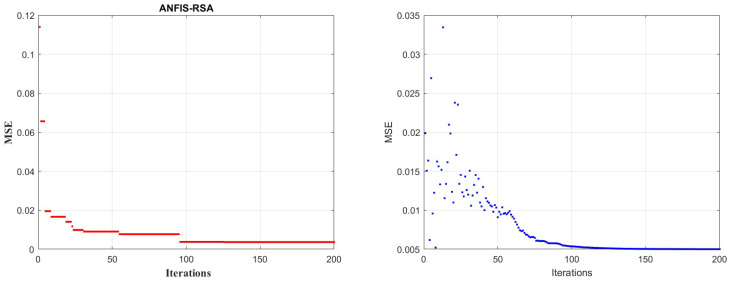
Convergence speed of trained ANFIS-RSA vs. ANFIS-CESBAS models for India.

In addition, the MSE performance of ANFIS-CESBAS has been presented in Figure 12.

**Figure 13:**
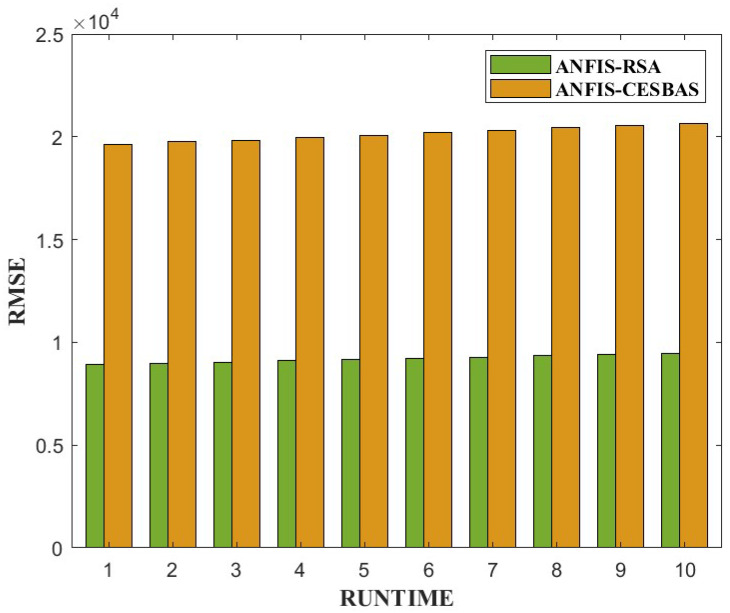
Comparative study of ANFIS-RSA vs. ANFIS-CESBAS of RMSE values on 10 runs.

**Figure 14:**
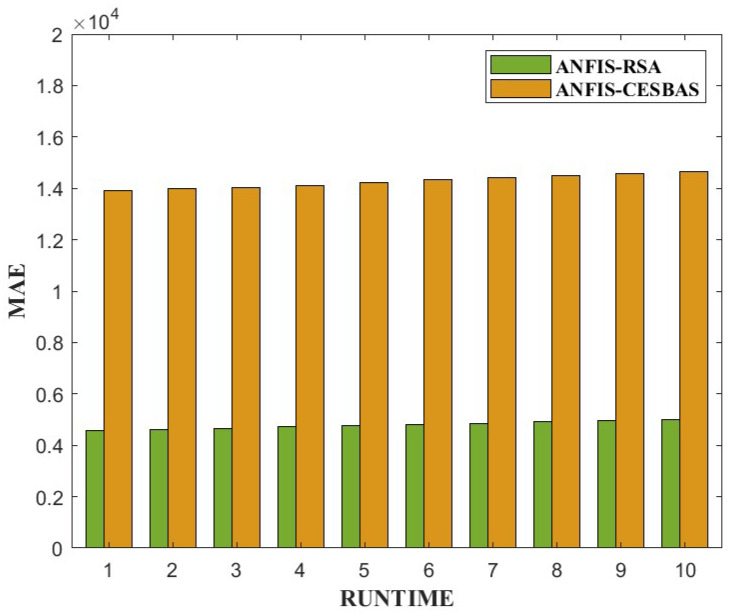
Comparative analysis of ANFIS-RSA vs. ANFIS-CESBAS of MAE values on 10 runs.

Furthermore, we conducted 10 independent runs to compare the performance. Figure 13 and Figure 14 exhibit bar plots of RMSE and MAE values over 10 runs of ANFIS-RSA and ANFIS-CESBAS.

**Figure 15:**
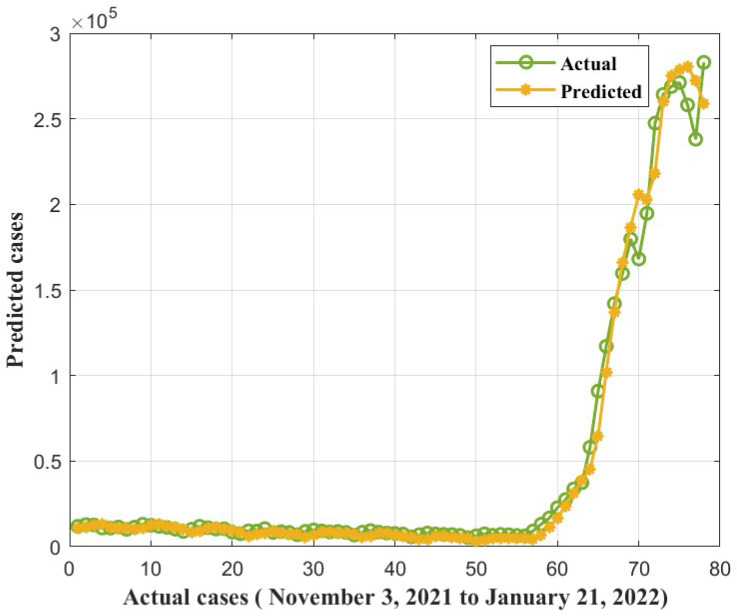
COVID-19 cases forecasted by ANFIS-RSA from 3 November 2021 to 21 January 2022.

**Figure 16:**
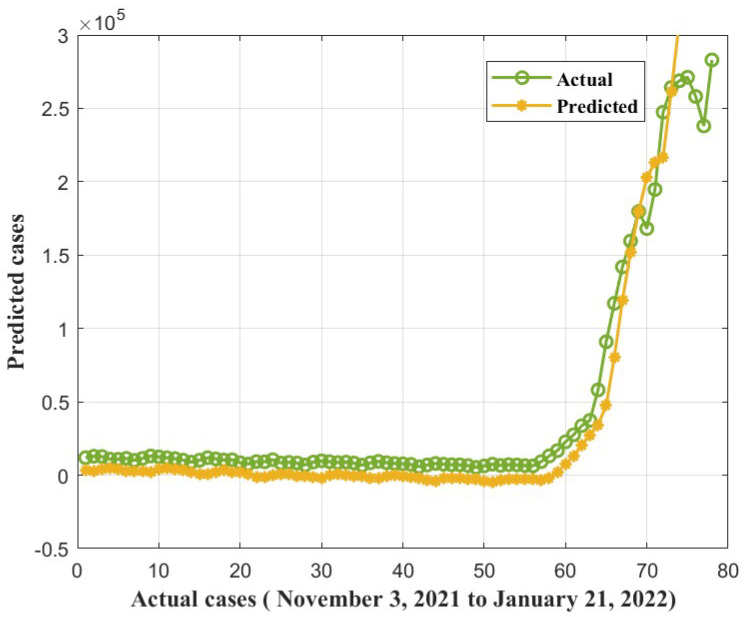
COVID-19 cases forecasted by ANFIS-CESBAS from 3 November 2021, to 21 January 2022.

The proposed hybrid ANFIS-RSA predictions for COVID-19 infections plotted against the actual cases from WHO between 3 November 2021, and 21 January 2022, in Figure 15, as well as the predictions of recently improved model ANFIS-CESBAS, appear in Figure 16.

### 5.3. Model Discussion

A realistic approach to estimating COVID-19-positive cases might assist in the formulation of a strategy for reducing positive infections. The two distinct COVID-19 datasets from China and India are gathered from the WHO in order to evaluate COVID-19 cases. Positive COVID-19 cases are collected in China from 21 January 2020, through 18 February 2020, and in India from 3 November 2021, through 21 January 2022. As seen in Figure 4, there are very few differences in the data from China. However, there are significant variations in the number of positive cases from India, as depicted in Figure 11. In general, increasing the level of uncertainty in the data decreases the accuracy of the prediction model. Here are some examples of how ML techniques were used to make COVID1-19 forecasts for a wide range of countries. Chowdhury et al. [50] applied both ANFIS and LSTM networks to simulate COVID-19 in Bangladesh, and the two methods were compared. Jithendra and Sharief-Basha [51] proposed a COVID-19 prediction model based on the Sugeno Adaptive Neuro-Fuzzy Inference System (SANFIS), and it achieved exceptional performance. By combining the ANFIS and virus optimization algorithm (VOA), Behnood et al. [21] also look at how different climate parameters and the number of people in an area affect the spread of COVID-19.

In contrast to existing models for predicting COVID-19 cases, this study developed a novel technique for predicting COVID-19 by integrating ANFIS and RSA optimization. Using the RSA, the relevant ANFIS model parameters (premise and consequent) are selected. The proposed ANFIS-RSA model is implemented using WHO data on COVID-19 cases in China to compare its robustness against ANFIS-CESBAS. The ANFIS-RSA performance for China is presented in Figure 5. In addition, a comparison study of existing methods with respect to the statistical metrics indicated in Table 2 was performed. From Table 2, it can be seen that the ANFIS-RSA obtains the best statistical metric values, indicating that it outperforms other methods. Figure 6 and Figure 7 provide a visual comparison of ANFIS-RSA and ANFIS-CESBAS for the RMSE and MAE metrics using bar charts. For this comparison, both techniques were executed 10 times each. As can be seen from the visual depiction of the data for ANFIS-RSA and ANFIS-CESBAS in Figure 10, ANFIS-RSA predicts the total number of cases in China with much more accuracy than ANFIS-CESBAS. According to the experimental findings, the ANFIS-RSA is the winner in practically all assessment tests and it outperformed existing methods in the literature. Lastly, the authors have trained the presented ANFIS-RSA model using recent data from the WHO about confirmed cases in India. The objective was to highlight the effectiveness of ANFIS-RSA and make very accurate predictions about the number of probable cases. The estimated cases of India using ANFIS-RSA are displayed against the observed ones in Figure 15. Similarly, Figure 16 depicts ANFIS-CESBAS results.

In comparison with all other approaches that have been considered in the study, the proposed ANFIS-RSA shows better performance when average results are taken into account. Similarly, only ANFIS-CESBAS achieved the highest $R^{2}$ metric comparison in the China dataset. On the other hand, the third-best approach was ANFIS-FPPASSA in simulations with the China dataset, which managed to outperform CESBAS-ANFIS in MAE and RMSRE results. ANFIS-RSA outperformed CESBAS by achieving a better balance between intensification and diversification. However, ANFIS-CESBAS performed poorly in the COVID-19 simulations in India.

## 6. Conclusions

The primary goal of our research is to enhance the ANFIS model to make it more accurate in optimizing non-linear problems and time-series predictions. As the most difficult and challenging aspect of ML techniques is optimizing parameters, in order to achieve accurate results, the RSA was incorporated into ANFIS. The proposed approach (ANFIS-RSA) was simulated in MATLAB 2020b, and comparisons have been done against previously developed methods. This hybrid technique was used to examine the effectiveness of the technique using COVID-19 data from India and China. Since COVID-19 has been the most prevalent disease that has posed a threat to people all over the world for the past three years, it is necessary to forecast the disease in order to accurately diagnose it. Moreover, the suggested approach may be useful for any classification and prediction and is not confined to COVID-19 predictions. The main focus of this article is to enhance the forecasting precision of future COVID-19 cases. In earlier studies, many researchers have managed to achieve accuracy in predicting COVID-19 disease; however, the suggested technique ANFIS-RSA outperforms the existing methods. The experimental and simulation results revealed that the model is more efficacious in anticipating the new COVID-19 infections and attained smaller RMSE, MAE, MAPE, and RMSRE values and the best coefficient of determination close to 1 for India and China. In addition, the speed of convergence of the ANFIS-RSA for China and India was visualized in Figure 5 and Figure 12, respectively. Finally, it is concluded that the suggested approach is quite beneficial for accurately predicting any time series data in a short period of time. Future studies will focus on improving the ANFIS models by introducing metaheuristic algorithms and modifying the RSA. In addition, we are focussing on inventing nature-inspired algorithms and integrating them with ANFIS to make a hybrid ensemble machine-learning model.

## Figures and Tables

**Table 3:** Comparative study of ANFIS-RSA vs. ANFIS-CESBAS for the testing dataset.

Enhanced Models	RMSE	MAE	MAPE	RMSRE	$R^{2}$
ANFIS-CESBAS	3618.7939	2441.429	0.0409	0.0581	0.8867
**ANFIS-RSA**	**3277.8871**	**2424.857**	**0.0408**	**0.0535**	**0.9071**

**Table 4:** Comparative study of ANFIS-RSA vs. ANFIS-CESBAS for the training dataset.

Enhanced Models	RMSE	MAE	MAPE	RMSRE	$R^{2}$	Time
ANFIS-CESBAS	19645.62	13924.641	0.9179	0.1056	0.9388	23.41
**ANFIS-RSA**	**8921.608**	**4570.359**	**0.2252**	**0.3338**	**0.9874**	**23.97**

**Table 5:** Comparative study of ANFIS-RSA vs. ANFIS-CESBAS for the testing dataset.

Enhanced Models	RMSE	MAE	MAPE	RMSRE	$R^{2}$
ANFIS-CESBAS	29344.008	23059.7	0.7121	1.6298	0.9041
**ANFIS-RSA**	**17281.4733**	**13240.4**	**0.1373**	**0.1888**	**0.9667**

## Data Availability

Data for this research was taken from publicly available repositories of WHO and government agencies.
